# Ten-Year Follow-Up of Taliglucerase Alfa in Type 1 Gaucher Disease: Real-World Evidence from Albania

**DOI:** 10.3390/jcm14197015

**Published:** 2025-10-03

**Authors:** Paskal Cullufi, Virtut Velmishi, Erjon Troja, Sonila Tomori, Ermira Dervishi, Gladiola Hoxha, Marjeta Tanka, Polikron Pulluqi, Adela Perolla, Entela Basha, Arben Ivanaj, Eda Jazexhiu, Mirela Tabaku

**Affiliations:** 1 Pediatric Department, University Hospital Centre “Mother Teresa”, 1000 Tirana, Albania; paskalcullufi@gmail.com (P.C.); tutimodh@yahoo.com (V.V.); s_tomorius@yahoo.com (S.T.); miradervishi45@gmail.com (E.D.); ola.hoxha1@gmail.com (G.H.); 2 Department of Pharmacy, University Hospital Centre “Mother Teresa”, 1000 Tirana, Albania; erjontroja@gmail.com; 3 Department of Imaging, University Hospital Centre “Mother Teresa”, 1000 Tirana, Albania; marjeta.tanka@yahoo.com; 4 Department of Hematology, University Hospital Centre “Mother Teresa”, 1000 Tirana, Albania; ppulluqi@yahoo.com (P.P.); adelaperolla@gmail.com (A.P.); aivanaj@yahoo.fr (A.I.); 5 Department of Neuroscience, University Hospital Centre “Mother Teresa”, 1000 Tirana, Albania; entelabasha@yahoo.com; 6 Neonatology Department, “Hygeia” Hospital, Tirana, Albania, University of Medicine, 1000 Tirana, Albania; edapostoli@hotmail.com; 7 Pediatric Department, Genetic Service, University of Medicine, 1005 Tirana, Albania

**Keywords:** Gaucher disease type 1, taliglucerase alfa, enzyme replacement therapy, Lyso-Gb1, chitotriosidase, imiglucerase switch

## Abstract

**Background/Objectives**: Gaucher disease type 1 is an autosomal recessive lysosomal storage disorder caused by pathogenic variants in the *GBA*1 gene. Although enzyme replacement therapy has improved patient outcomes, there is limited long-term real-world data on taliglucerase alfa. This study aimed to evaluate the long-term efficacy and safety of taliglucerase alfa in both treatment-naïve and previously treated patients with Gaucher disease type 1 over a 10-year period. **Methods**: This prospective, single-centre cohort study involved 29 patients (13 treatment-naïve and 16 previously treated with imiglucerase) who received taliglucerase alfa from 2015 to 2024. Clinical, hematological, visceral, skeletal, and biochemical parameters were assessed at baseline and at 12, 60, and 120 months. Biomarkers included chitotriosidase and glucosylsphingosine. Safety was evaluated through adverse event reporting and anti-drug antibody testing. **Results**: Hemoglobin and platelet counts improved or remained stable in all patients. By 60 months, liver volume had normalised in treatment-naïve patients (mean reduction: 23.1%), while spleen volume had decreased by up to 47.3%. Lyso-Gb1 levels decreased by 86.1% in patients who had not previously received treatment and by 59.5% overall, with a strong correlation to adherence. Bone mineral density improved in most cases. 137 adverse events were reported, 24% of which were mild infusion-related reactions. Anti-drug antibody developed in two patients, including one with a reduced therapeutic response. **Conclusions**: Taliglucerase alfa offers sustained long-term clinical, hematological and biochemical benefits in both treatment-naïve and previously treated Gaucher disease type 1 patients, with a favorable safety profile. Glucosylsphingosine proved to be a highly sensitive biomarker for monitoring therapeutic efficacy and detecting treatment response.

## 1. Introduction

Gaucher disease (GD) is a rare autosomal recessive lysosomal storage disorder caused by biallelic pathogenic variants in the *GBA1* gene, which encodes the lysosomal enzyme β-glucocerebrosidase [1]. A deficiency of this enzyme leads to the accumulation of glucosylceramide and its more cytotoxic derivative, glucosylsphingosine (Lyso-Gb1), within macrophages of the reticuloendothelial system. This progressive storage process results in the infiltration of organs such as the spleen, liver, bone marrow and skeletal tissue [2,3,4], underlying most clinical manifestations [2]. In addition to substrate accumulation, other mechanisms play an important role in GD pathogenesis, including chronic immune activation, cytokine release, dysregulated inflammatory responses and impaired autophagy [5,6,7]. GD is characterized by extensive allelic heterogeneity, with over 860 *GBA1* variants reported to date [8]. GD presents with a broad phenotypic spectrum ranging from severe neonatal disease to asymptomatic adult-onset forms [9]. Traditionally, GD has been categorized into three clinical subtypes based on the presence and severity of neurological involvement. However, it is increasingly recognized as a continuum phenotypic [7,10] influenced by genetic, epigenetic, and environmental modifiers [3].

Enzyme replacement therapy (ERT) has significantly improved the prognosis of patients with type 1 Gaucher disease (GD1), effectively reducing organomegaly, reversing cytopenias and preventing skeletal complications [7,11,12,13]. Since its introduction in the early 1990s, therapeutic advances have led to the development of several ERT options and substrate reduction therapies. In parallel, biomarker research has established Lyso-Gb1 as a highly sensitive diagnostic and monitoring tool that reflects disease burden and treatment response [14,15,16,17]. There are currently three enzyme replacement therapies (ERTs) available for Gaucher disease: imiglucerase (produced by Sanofi/Genzyme in Chinese hamster ovary cells); velaglucerase alfa (produced by Takeda/Shire in cultured human cells); and taliglucerase alfa (produced by Pfizer/Protalix in plant cells), which is the first recombinant therapeutic protein of its kind to be approved for human use [12,18,19]. Taliglucerase alfa (TGa) is produced in genetically modified carrot cells. Unlike enzymes derived from mammalian cells, TGa undergoes natural glycosylation with terminal mannose residues, eliminating the need for glycan modifications after production to enable uptake by Gaucher cells. Although its plant origin may slightly increase the risk of infusion-related adverse reactions, TGa offers a cost-effective and scalable manufacturing process [12,20,21]. It has been approved for the long-term treatment of type 1 Gaucher disease (GD1), and in some countries, including Albania, for managing visceral and hematological manifestations in type 3 GD patients [12,19,22]. Clinical trials have demonstrated that TGa has a similar efficacy with other ERT in improving visceral, hematological and skeletal outcomes [23,24,25,26]. However, real-world long-term follow-up data remain limited, particularly in genetically unique populations.

This study presents prospective, real-world data on the safety and efficacy of taliglucerase alfa over a ten-year period in a cohort of Albanian patients with type 1 Gaucher disease, including treatment-naïve individuals and those switched from imiglucerase. The aim is to address current gaps in the literature by providing the longest observational follow-up of TGa therapy to date, with detailed clinical, biochemical, imaging and biomarker outcomes.

## 2. Materials and Methods

### 2.1. Study Design and Participants

This prospective cohort study was conducted at a specialized Gaucher unit of the ‘Mother Teresa’ University Hospital Centre (UHC) in Tirana, Albania, from January 2015 to December 2024. Approval for the study was obtained from the National Ethics Committee. Written informed consent was obtained from all participants, or from their legal guardians in the case of minors, prior to enrolment.

The study included patients diagnosed with type 1 Gaucher disease who had received treatment with taliglucerase alfa for at least one year. Both pediatric and adult patients were enrolled, including those who were treatment-naïve and those who had previously received treatment with imiglucerase. Patients were required to have a confirmed diagnosis of GD to be enrolled in the study, which could be established either biochemically by reduced glucocerebrosidase activity or genetically by *GBA*1 genotyping.

Data collection. The data included patient demographics, diagnoses, treatment details, hematological and visceral parameters, adverse events and relevant biomarkers. Liver and spleen volumes were measured annually using magnetic resonance imaging (MRI), computed tomography (CT), or three-dimensional (3D) ultrasound, while orthopedic imaging included X-rays, ultrasound, MRI, and CT.

A total of 29 patients diagnosed with GD1 were enrolled in the study. These patients were divided into two groups: [1] treatment-naïve patients and [2] patients who had previously been treated with imiglucerase.

TGa was administered via intravenous infusions every two weeks. For patients switched from imiglucerase, the same dose was maintained. For treatment-naïve patients, the initial dose ranged from 30 to 60 U/kg depending on clinical presentation and laboratory findings.

Clinical and laboratory data were prospectively recorded during routine follow-up visits and included the following: demographics; clinical history and physical examination; hematological and visceral parameters; bone imaging results; biomarkers; adverse events (AEs).

### 2.2. Efficacy Assessment

The efficacy of TGa was evaluated by monitoring several key parameters.

Hematological measures: hemoglobin concentration, platelet count.

Biomarkers: Chitotriosidase activity was measured every six months until 2019.

Plasma Lyso-Gb1 levels have been monitored biannually since 2016. To investigate potential correlations with *GBA*1 genotypes, we analyzed levels in patients who had not received treatment and conducted genotype-based comparisons.

Organ volumes: Liver and spleen volumes were assessed using magnetic resonance imaging (MRI). If an MRI scan was unavailable or declined, computed tomography (CT) was used as an alternative. Organ volumes were expressed as multiples of normal (MN), with normal values defined as 25 mL/kg of body weight for the liver and 2 mL/kg of body weight for the spleen.

At baseline, spleen volume was assessed in treatment-naïve patients, who were categorized into three groups based on the degree of splenomegaly: mild (<5 MN), moderate (5–15 MN), and severe (>15 MN).

Bone mineral density (BMD) was assessed annually via dual-energy X-ray absorptiometry (DXA) at the lumbar spine and femoral neck. Results were reported as Z-scores and classified as follows: Normal: Z-score > −1.0; Osteopenia: Z-score between −1.0 and −2.5. Osteoporosis: Z-score < −2.5.

Data for each parameter were analyzed at baseline and after 12, 60 and 120 months of treatment.

### 2.3. Safety Assessment

Safety was monitored by adverse events (AEs) and the formation of antidrug antibodies (ADAs). AEs were categorized as infusion-related or non-infusion-related and as mild, moderate or serious. Serious adverse events (SAEs) were defined as events causing death, life-threatening illness, hospitalization, or persistent disability. Anti-drug antibodies were measured every six months for the first three years of TGa treatment only.

### 2.4. Statistical Analysis

Data were summarized by group (treatment-naïve, previously treated, and the total cohort) using the following: Continuous variables: mean, standard deviation (SD) and range. Categorical variables were summarized using counts and percentages. A Student’s *t*-test was used to evaluate changes from baseline. Analyses were performed using IBM SPSS Statistics version 26.0.

## 3. Results

### 3.1. Patient Characteristics

Twenty-nine patients with type 1 Gaucher disease were included in the study. The cohort consisted of 18 males and 11 females (a ratio of 1.6:1). Sixteen patients had previously received imiglucerase treatment, while thirteen were treatment-naïve.

The mean age at diagnosis was 36.15 ± 22.82 years for treatment-naïve patients and 13.69 ± 10.01 years for those who had switched treatment. The corresponding mean ages at disease onset were 26.85 ± 17.55 years and 10.67 ± 9.20 years, respectively. These figures indicate an average overall delay of about 6 years, with respective delays of 9.30 and 3.02 years for treatment-naïve and switched patients.

The significant delay observed in treatment-naïve patients is likely related to the milder clinical manifestations in this group.

In our cohort, 65.5% of patients (19 out of 29) carried the complex genotype p.[Asn409Ser];[His255Gln;Asp448His]. This genotype has been reported almost exclusively in Albanian and neighbouring populations, suggesting a regional founder effect. The p.[Asn409Ser];[other] genotype was identified in eight patients (27.6%), including two patients with the p.Leu483Pro variant (formerly L444P), which is the most prevalent *GBA*1 mutation worldwide, underscoring the distinct mutation spectrum of Gaucher disease in Albania. The remaining two patients, who were siblings, had very rare genotypes: p.Asp448His, p.Leu422Profs*4 and p.Arg87Trp. This family comprises seven affected individuals across three generations. This genetic profile highlights the importance of population-specific counselling and diagnostic strategies, and supports the hypothesis that historical founder effects have shaped the unique genetic landscape of Gaucher disease in this region.

The most common clinical manifestations were splenomegaly in 24 patients (82.75%) and hepatomegaly in 13 patients (44.82%). Thrombocytopenia was observed in 11 individuals (37.93%), while bone pain affected seven patients (24.13%).

### 3.2. Treatment Efficacy

- Hemoglobin concentration

**Figure 1:**
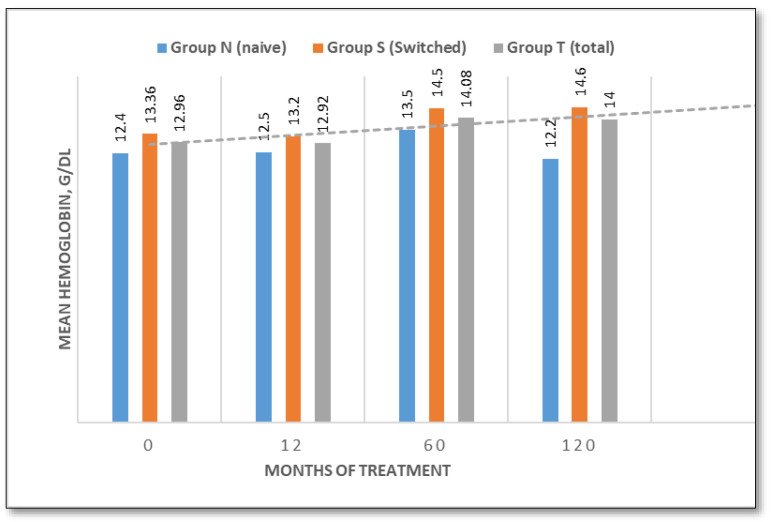
Hemoglobin concentration changes over time (naive/switched/total patient groups).

At baseline, the mean hemoglobin (Hb) concentration was 12.4 g/dL (range 10.7–14.8 g/dL) in the treatment-naïve group, and 13.36 g/dL (range 11.6–15.5 g/dL) in the switched group. In the treatment-naïve group, hemoglobin increased to 12.5 g/dL (+0.8%) at 12 months, to 13.5 g/dL (+9.7%) at 60 months and stabilized at 12.2 g/dL (−1.6%) by 120 months. In the switched group, Hb decreased slightly at 12 months (to 13.2 g/dL; −1.2%), followed by increases at 60 months (to 14.5 g/dL; +7%) and 120 months (to 14.6 g/dL; +8.5%) (Figure 1).

- Platelet Count

**Figure 2:**
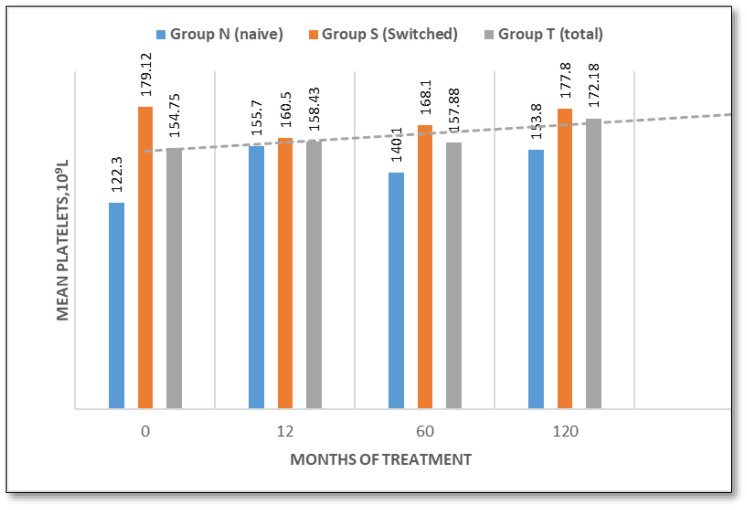
Platelet value evaluation over time (naive/switched/total patient groups).

Baseline mean platelet (Plt) counts were significantly lower in the naïve group (122.3 × 10^3^/μL; range 37–215) than in the switched group (179.12 × 10^3^/μL; range 104–369). In patients who were treatment-naïve, the platelet count increased to 155.7 × 10^3^/μL (27.3%) at 12 months and to 168.12 × 10^3^/μL (37.5%) at 60 months. It then decreased slightly to 153.8 × 10^3^/μL (13%) at 120 months. Patients who switched treatment experienced a modest decline to 160.5 × 10^3^/μL (−10.4%) at 12 months, subsequently recovering to 177.83 × 10^3^/μL at 60 months (+6.2%). Values remained stable at 177.8 × 10^3^/μL at 120 months (−0.74%), (Figure 2). Despite this, variations in platelet count were observed in individual patients during follow-up.

- In parallel, we evaluated the progression of platelet counts in treatment-naïve patients who presented with thrombocytopenia at baseline. The mean platelet count at baseline was 90.85 × 10^9^/L, which increased to 132.5 × 10^9^/L (an improvement of approximately 30%) by the end of the monitoring period.
- Hepatic volume

**Figure 3:**
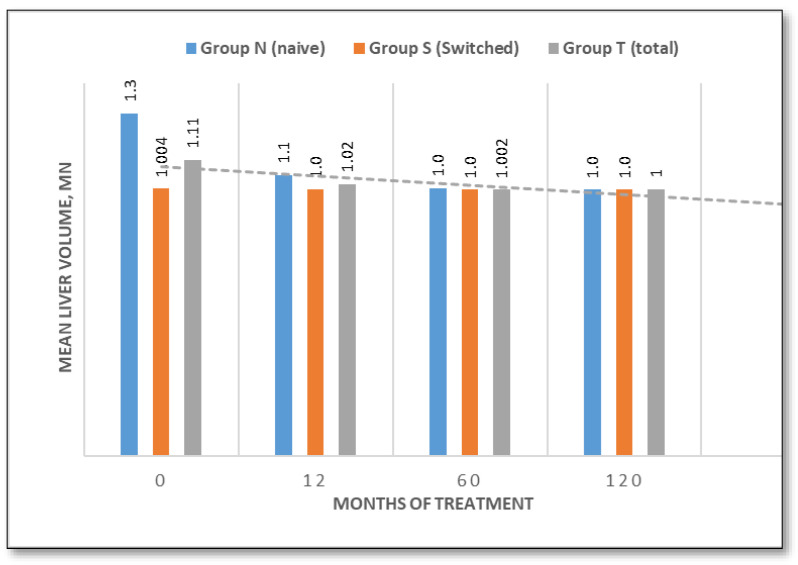
Liver volume evaluation over time (naive/switched/total patient groups).

Liver volume, expressed as multiples of normal, was 1.3 MN (range 1.0–2.1 MN) in the naïve group and 1.004 MN (range 1.0–1.07 MN) in the switched group at baseline. Patients in the naïve group demonstrated progressive decreases to 1.1 MN (−15.4%) at 12 months and 1.0 MN (−23.1%) at 60 months, with stable volumes thereafter. Switched patients maintained stable liver volumes near normal levels (1.0 MN) throughout (Figure 3). Final liver volumes normalised to 1.0 MN across all groups.

- Splenic volume

**Figure 4:**
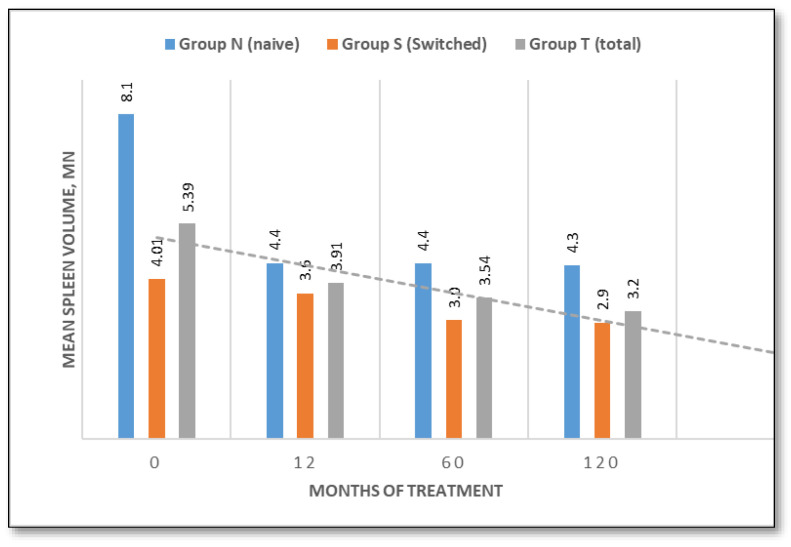
Spleen volume evaluation over time (naive/switched/total patient groups).

Initial spleen volumes were significantly larger in naïve patients (8.1 MN, range 2.5–18.06 MN) than in the switched group (4.01 MN, range 1.33–8.78 MN). In the naïve group, volumes decreased to 4.4 MN (46%) at 12 and 60 months and declined further to 4.3 MN (47.3%) by 120 months. In the switched group, volumes decreased from 4.01 MN to 3.6 MN after 12 months (10.3%), to 3.0 MN after 60 months (25.2%), and to 2.9 MN after 120 months (27.7%), (Figure 4).

At baseline, spleen volume was assessed, and naïve patients were categorized into three groups based on the degree of splenomegaly: one patient had severe splenomegaly (>15 MN), seven patients had moderate splenomegaly (5–15 MN), and three patients had mild splenomegaly (<5 MN). Additionally, two patients had previously undergone splenectomy. Over the course of treatment, spleen volume progressively decreased. After 5 years of therapy, only 3 out of 11 patients remained with moderate splenomegaly, while the others improved to mild forms. By the end of the study period, only one patient remained in the moderate category, with a spleen volume of 6.6 MN.

- Chitotriosidase activity

**Figure 5:**
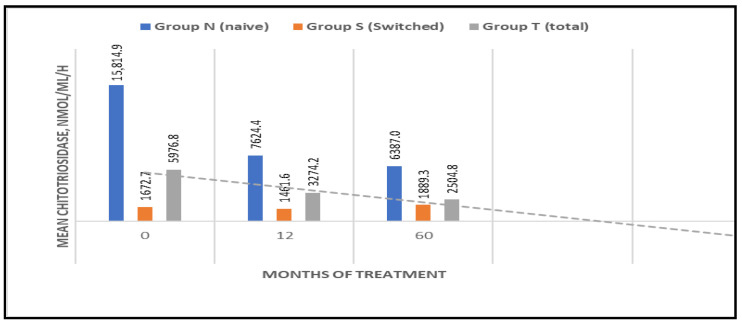
Chitotriosidase value evaluation over time (naive/switched/total patient groups).

Baseline chitotriosidase activity was significantly higher in naïve patients (mean: 15,814.9 nmol/mL/h; range: 868–47,280). A substantial decline was observed post-treatment: 7624.4 nmol/mL/h (52%) at 12 months, and 6387.04 nmol/mL/h (60%) at 60 months. In the switched group, baseline activity was lower (mean: 1672.7 nmol/mL/h; range: 70–4260) and declined modestly to 1461.6 nmol/mL/h (6.7%) after 12 months. However, an increase to 1889.34 nmol/mL/h (+11.5%) was observed in two patients receiving corticosteroid therapy for asthma after 60 months (Figure 5).

- Lyso-Gb1 Biomarker

At baseline, treatment-naïve patients exhibited markedly elevated Lyso-Gb1 levels (mean: 366.1 ng/mL; range: 72–1090 ng/mL), whereas switched patients had significantly lower values (mean: 82.2 ng/mL; range: 27–229 ng/mL). In naïve patients, levels dropped to 203.2 ng/mL (44.5%) after 12 months, continuing to decrease to 78.5 ng/mL (78.56%) after 60 months, and reaching 51.07 ng/mL (86.1%) after 120 months.

Correlation analysis revealed that individuals with the complex p.[Asn409Ser];[His255Gln;Asp448His] genotype had significantly lower Lyso-GB1 concentrations (251 ng/mL) at baseline than those with other genotypes (767 ng/mL, *p* < 0.01). This trend was sustained over time, with Lyso-GB1 levels remaining lower after 10 years in patients with the p.[Asn409Ser];[His255Gln;Asp448His] genotype (50.9 ng/mL) than in those with other genotypes (126.7 ng/mL). These results suggest that the p.[Asn409Ser];[His255Gln;Asp448His] genotype may be linked to a less severe biochemical presentation of the disease.

**Figure 6:**
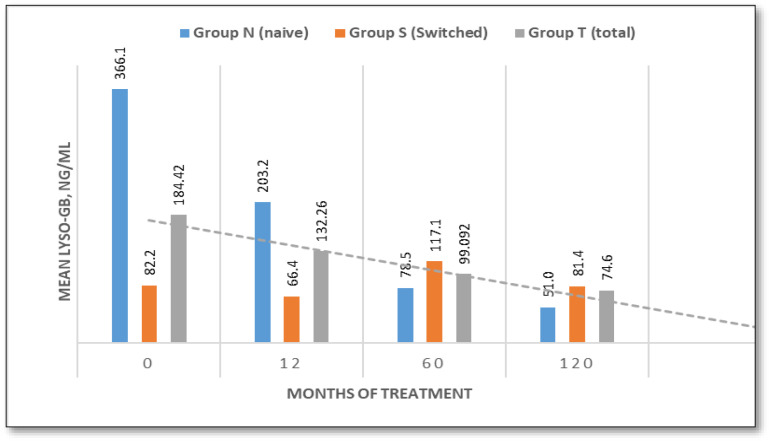
Lyso-Gb1 value evaluation over time (naive/switched/total patient groups).

In the switched group, lyso-Gb1 levels decreased from 88.2 ng/mL to 66.4 ng/mL after 12 months (−19.2%). However, an increase of 43.3% was observed at 60 months compared to the 12-month level due to therapy interruption. Upon resumption of therapy, levels declined by 37% (from 117.1 ng/mL to 81.43 ng/mL) at 120 months. (Figure 6).

**Figure 7:**
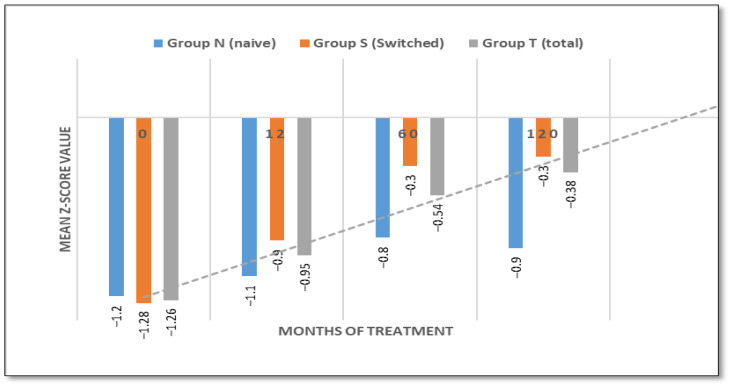
Z-score value evaluation over time (naive/switched/total patient groups).

- Bone Mineral Density (Z-score):

Baseline lumbar spine BMD Z-scores were −1.2 for naïve patients, −1.28 for switched patients and −1.26 for all patients. Naïve patients achieved Z-scores of −0.8 after 60 months and 0.9 after 120 months. Patients who had switched treatment improved from a Z-score of −1.28 to −0.3 after 120 months. Both groups normalized the Z-score after 60 months (see Figure 7). However, one patient exhibited refractory osteopenia (Z-score of approximately −2) throughout the study, despite improvements in hematology and visceral manifestations.

At the end of the study, despite overall improvements, eight patients (four from each group) presented with osteopenia, with Z-scores ranging from –1 to –2. This finding is consistent with existing data indicating that the treatment of bone disease with enzyme replacement therapy remains a clinical challenge.

### 3.3. Safety Profile

A total of 137 adverse events (AEs) were recorded among 24 patients (82.7%). Of these, 52 (37.8%) were infusion-related and were reported by seven patients (24%), primarily during the first infusions. The most common AEs were urticaria, lip oedema, and pruritus. One severe infusion reaction occurred in an 80-year-old male following a two-month interruption to treatment, presenting with hypotension, tachycardia, a weak radial pulse, chills, respiratory distress and systemic symptoms.

One patient experienced delayed infusion reactions: he was asymptomatic immediately post-infusion, but the following day he developed flu-like symptoms, including nausea, body and muscle pain, chills, and fever. This pattern recurred three times following respective drug administrations, but the patient initially did not report the symptoms, attributing them to viral infections.

Non-infusion-related adverse events (AEs) were noted in 17 patients (58.6%), including three severe events. One patient was hospitalized and underwent surgery for cholelithiasis. Two patients died: one from a brain tumor and the other from worsening neurodegeneration associated with Parkinson’s disease. Frequent non-infusion-related AEs included rhinopharyngitis (16.8%), SARS-CoV-2 infection (5.1%), and psychiatric symptoms (4.3%) (Table 1).

Routine anti-drug antibody (ADA) testing identified two seropositive patients. One of these patients had a low-titer IgG ADA, which was not associated with clinical changes. The other patient had a high-titer IgG ADA and concomitant autoimmune thyroiditis. This patient, a woman aged around 40, exhibited a reduced response to therapy, with increasing lyso-Gb1 and chitotriosidase levels and a decreased platelet count.

## 4. Discussion

This ten-year longitudinal evaluation of TGa in patients with type 1 Gaucher disease demonstrates sustained efficacy in terms of hematological manifestations, hemoglobin and platelet count. Patients either exhibited an improvement in baseline values throughout the study, particularly in naïve patients, or remained stable. These data are consistent with prior studies confirming the hematological benefits of TGa in the treatment of GD1 patients [12,18,24,25]. Hepatosplenomegaly is one of the most consistent clinical findings in GD1 patients. At baseline, liver volume was mildly elevated in untreated patients (1.30 MN), but normal in the switched group (1.004 MN), likely reflecting the efficacy of prior treatment. Liver volumes normalized in naïve patients by month 60 and remained stable thereafter. Spleen volumes declined significantly by 47.3% in naïve patients and by 27.7% in switched patients over 120 months, which reinforces the long-lasting visceral benefits of TGa and confirms data from previous studies [12,18,19,26,27].

Chitotriosidase activity, a marker of macrophage activation, was markedly elevated at baseline in naïve patients, declining by 52% at 12 months and by 60% at 60 months. In patients who had undergone treatment, baseline levels were lower, with a modest reduction of 6.7% observed at 12 months. However, a subsequent increase of 11.5% was noted at 60 months, probably due to an increase in chitotriosidase activity in two individuals undergoing corticosteroid therapy, which is reported to influence chitotriosidase activity [28]. This underlines the importance of considering comorbidities and drug interactions when interpreting biomarker dynamics, or a worsening of other parameters of the disease, such as platelet count, hemoglobin concentration, bone disease, or organ volume. However, it also underscores the limited utility of chitotriosidase as a GD biomarker, given that it is not directly implicated in the pathogenesis of the disease, but rather reflects the activation of macrophages following glucocerebroside uptake [14,15,16]. Furthermore, chitotriosidase is an unreliable biomarker in individuals carrying null alleles of the CHIT1 gene, thus limiting its applicability in monitoring GD.

In contrast, Lyso-Gb1 is a sensitive and specific marker for diagnosing GD and monitoring disease burden [14,15,29,30]. Naïve patients demonstrated a significant decrease in Lyso-Gb1 levels, dropping from 366.1 ng/mL to 51.07 ng/mL (an 86.1% reduction) over 120 months. In patients who had switched therapy, an initial reduction of 19.2 was observed at 12 months, followed by a transient increase at 60 months due to a three-month interruption to treatment. During this time, biomarker levels tripled. Following the resumption of therapy, a 37% decline was observed, highlighting Lyso-Gb1′s responsiveness to therapeutic adherence.

These findings reinforce Lyso-Gb1′s sensitivity to treatment continuity and highlight its potential utility in monitoring compliance and therapeutic efficacy. These data also reveal that monitoring Lyso-Gb1 enables the early detection of absent or insufficient treatment before clinical consequences arise [29,30]. Consequently, any unexpected increase in Lyso-Gb1 levels should prompt a clinical evaluation to identify the underlying cause. Due to its high sensitivity, Lyso-Gb1 has also been reported to be useful for monitoring asymptomatic, untreated patients [31] and those diagnosed by newborn screening [32].

The significant diagnostic delay of around ten years observed in our treatment-naïve patients underscores the importance of more frequent use of the Lyso-Gb1 biomarker to enable earlier diagnosis, especially in patients exhibiting clinical symptoms indicative of GD, such as thrombocytopenia and splenomegaly.

Osteopenia and osteoporosis are prevalent complications of GD1 that contribute to pain, an increased risk of fracture, and a reduced quality of life [7,11,33,34,35]. Improvements in bone mineral density (BMD) were observed in both patient groups. Z-scores increased from −1.2 to −0.9 in naïve patients, and from −1.28 to −0.3 in switched patients, due to the longer treatment duration. These findings support previous reports indicating that TGa has a beneficial impact on skeletal remodeling [11,26,36]. Despite the overall improvements, one patient remained refractory to treatment-related improvements in BMD, with persistently low Z-scores (of approximately −2). Interestingly, this was associated with a high level of Lyso-GB1, despite favorable trends in hemoglobin concentration and platelet counts. This patient was found to carry the p.Asn409Ser/p.Ser147Leu genotype (legacy N370S/S146L) genotype, a rare and potentially more severe variant in the Albanian population.

Conversely, this case highlights the variability in skeletal responses to ERT and reflects the multifactorial nature of bone disease in GD, suggesting that ERT treatment alone may not be sufficient for complete resolution [7,37].

Seven patients (24%) experienced infusion-related adverse events (AEs), primarily during the initial infusions, which were generally mild to moderate in severity. This is consistent with the safety profile observed in clinical trials and observational cohorts [12,18,19,20,22,23,24,27]. However, three patients reported AEs several months or years after starting treatment. Notably, one patient experienced a severe infusion-related reaction. This event was managed in a hospital setting and did not have any long-term consequences; the patient subsequently discontinued the TGa. Another patient developed delayed infusion reactions characterized by flu-like symptoms 24–72 h after infusion, which is a pattern that has not been reported previously with taliglucerase alfa [38]. These reactions warrant further investigation as potential delayed hypersensitivity syndromes.

Non-infusion-related AEs included common infections (e.g., rhinopharyngitis and SARS-CoV-2 infection), as well as psychiatric symptoms (e.g., anxiety and depression), occurring in six patients (4.3%). This is a noteworthy finding as such events are rarely described in GD cohorts. However, they may reflect the chronic nature of the disease and its psychosocial impact. ADA formation is less commonly associated with clinical impact in GD than in other lysosomal storage disorders [39,40]. Anti-taliglucerase alfa antibodies were identified in two cases in our cohort. One patient with low-titer IgG ADA remained clinically stable. The other patient was a 40-year-old woman with autoimmune thyroiditis who developed a high ADA titer, which was associated with lower treatment efficacy as evidenced by increasing lyso-Gb1 and chitotriosidase levels and a decreased platelet count. Switching therapy led to an improvement in the level of biomarkers and the platelet count. This case highlights that ADA formation, particularly in the context of autoimmune comorbidities, can compromise treatment efficacy and necessitate personalized monitoring.

### Limitations of the Study

The relatively small size of the cohort may limit the generalizability of the findings. Furthermore, as with all real-world studies, the inherent limitations of observational data must be recognised. Despite these limitations, this study offers important insights into the long-term efficacy and safety of taliglucerase alfa therapy in a genetically distinct GD1 population, providing one of the most extensive real-world follow-ups to date.

## 5. Conclusions

This 10-year follow-up study provides robust, real-world evidence that TGa alfa is an effective and well-tolerated treatment for both treatment-naïve and previously treated patients with GD1. Sustained biochemical and clinical improvements were observed, particularly in treatment-naïve individuals. Monitoring lyso-Gb1 levels is essential for evaluating therapeutic response and guiding clinical decisions. While hematological and visceral responses were consistent, skeletal responses varied, emphasizing the need for targeted approaches in managing GD-related bone disease. These findings support taliglucerase alfa as a reliable long-term therapeutic option for patients with GD1.

## Figures and Tables

**Table 1:** Adverse events.

Adverse Events (AEs)	Patients, Nr (%)	Events, Nr (%)
Total AEs	24 (82.7%)	137 (100%)
Drug infusion-related AEs	7 (24%)	52 (37.8%)
Mild/Intermediate	6 (20.6%)	51 (37.2%)
Severe	1 (3.4%)	–
Most Frequent AEs (drug-related)		
Urticaria	5 (17.2%)	24 (17.8%)
Lip edema	3 (12.5%)	5 (3.6%)
Pruritus	3 (12.5%)	4 (2.9%)
Not drug-related AEs	17 (58.6%)	85 (62.0%)
Severe	3 (10.3%)	3 (2.1%)
Death	2 (6.8%)	2 (1.4%)
Most Frequent AEs (not drug-related)		
Rhinopharyngitis	12 (41.3%)	23 (16.8%)
COVID-19	7 (24.1%)	7 (5.1%)
Psychiatric disorders	6 (20.7%)	6 (4.3%)

## Data Availability

The data presented in this study are available on request from the corresponding author. The data are not publicly available due to privacy and ethical restrictions.

## Abbreviations

The following abbreviations are used in this manuscript:

ADA	Anti-Drug Antibody
AE	Adverse Event
BMD	Bone Mineral Density
CT	Computed Tomography
DBS	Dried Blood Spot
DXA	Dual-Energy X-ray Absorptiometry
ERT	Enzyme Replacement Therapy
FDA	Food and Drug Administration
GD	Gaucher Disease
GD1	Gaucher Disease Type 1
Hb	Hemoglobin
IgG	Immunoglobulin G
Lyso-Gb1	Glucosylsphingosine
MN	Multiples of Normal
MRI	Magnetic Resonance Imaging
Plt	Platelet Count
SAE	Serious Adverse Event
SD	Standard Deviation
SPSS	Statistical Package for the Social Sciences
TGa	Taliglucerase alfa
UHC	University Hospital Centre
